# Dual RNAseq highlights the kinetics of skin microbiome and fish host responsiveness to bacterial infection

**DOI:** 10.1186/s42523-021-00097-1

**Published:** 2021-05-07

**Authors:** J. Le Luyer, Q. Schull, P. Auffret, P. Lopez, M. Crusot, C. Belliard, C. Basset, Q. Carradec, J. Poulain, S. Planes, D. Saulnier

**Affiliations:** 1 Ifremer, IRD, Institut Louis-Malardé, Univ Polynésie Française, EIO, F-98719 Taravao, Tahiti, Polynésie Française; 2 MARBEC, Univ. Montpellier, Ifremer, IRD, CNRS, F-34200 Sète, France; 3 Virologie et Immunologie Moléculaires, Institut National de la Recherche Agronomique, Université Paris-Saclay, Jouy-en-Josas, France; 4 Univ Polynésie française, Ifremer, IRD, Institut Louis-Malardé, EIO, F-98702 Fa, ’a Tahiti, Polynésie Française; 5 Génomique Métabolique, Genoscope, Institut François Jacob, CEA, CNRS, Univ Evry, Université Paris-Saclay, 91057 Evry, France; 6 PSL Research University: EPHE-UPVD-CNRS, USR 3278 CRIOBE, Moorea, Polynésie Française; 7 Laboratoire d’Excellence “CORAIL,” USR 3278 CNRS-EPHE-UPVD CRIOBE, Perpignan, France

**Keywords:** Microbiome, Gene expression, 16S rRNA, Nanopore, *Tenacibaculum maritimum*, Co-infection

## Abstract

**Background:**

*Tenacibaculum maritimum* is a fish pathogen known for causing serious damage to a broad range of wild and farmed marine fish populations worldwide. The recently sequenced genome of *T. maritimum* strain NCIMB 2154^T^ provided unprecedented information on the possible molecular mechanisms involved in the virulence of this species. However, little is known about the dynamic of infection in vivo, and information is lacking on both the intrinsic host response (gene expression) and its associated microbiota. Here, we applied complementary omic approaches, including dual RNAseq and 16S rRNA gene metabarcoding sequencing using Nanopore and short-read Illumina technologies to unravel the host–pathogen interplay in an experimental infection system using the tropical fish *Platax orbicularis* as model.

**Results:**

We showed that the infection of the host is characterised by an enhancement of functions associated with antibiotic and glucans catabolism functions but a reduction of sulfate assimilation process in *T. maritimum*. The fish host concurrently displays a large panel of immune effectors, notably involving innate response and triggering acute inflammatory response. In addition, our results suggest that fish activate an adaptive immune response visible through the stimulation of T-helper cells, Th17, with congruent reduction of Th2 and T-regulatory cells. Fish were, however, largely sensitive to infection, and less than 25% survived after 96 hpi. These surviving fish showed no evidence of stress (cortisol levels) or significant difference in microbiome diversity compared with controls at the same sampling time. The presence of *T. maritimum* in resistant fish skin and the total absence of any skin lesions suggest that these fish did not escape contact with the pathogen, but rather that some mechanisms prevented pathogens entry. In resistant individuals, we detected up-regulation of specific immune-related genes differentiating resistant individuals from controls at 96 hpi, which suggests a possible genomic basis of resistance, although no genetic variation in coding regions was found.

**Conclusion:**

Here we focus in detail on the interplay between common fish pathogens and host immune response during experimental infection. We further highlight key actors of defence response, pathogenicity and possible genomic bases of fish resistance to *T. maritimum*.

**Supplementary Information:**

The online version contains supplementary material available at 10.1186/s42523-021-00097-1.

## Background

Pathogens remain a significant threat to biodiversity, livestock farming and human health [1]. Host–pathogen interactions rely on a complex balance between host defences and pathogen virulence. Through constant selective pressure, pathogens evolve mechanisms to overcome the host’s immune system. Reciprocally, the host adapts to counteract and limit pathogen virulence. Although changes in gene expression as a result of host–pathogen interactions appear to be common [2–4], the mechanisms involved often remain poorly understood. A more in-depth understanding of host–pathogen interactions could potentially improve our mechanistic understanding of pathogenicity and virulence, thereby defining novel preventive, therapeutic and vaccine targets [5].

Dual RNAseq sequencing fulfils the need to simultaneously assess the expression of both host and pathogen genes [6–8]. Studies applying this approach to fish bacterial infection systems have flourished recently and show promise for deciphering the complexity of host–pathogen interplay [9–11]. Yet, none of these studies have simultaneously explored dysbiosis and associated changes in microbiota. In nature, co-occurrence of multiple pathogen species (co-infection) is frequent. Species interactions can be neutral, antagonistic or facilitative and most often shape strain virulence plasticity, resulting in increased disease virulence [12–14]. Despite their commonness, remarkably few studies have explored such models, i.e. when a host interacts simultaneously with multiple pathogens during co-infection [15]. During tenacibaculosis outbreaks in *Platax*, *Tenacibaculum maritimum* burden is also commonly associated with other pathogen co-occurrences, namely *Vibrio* spp. [16]. Nevertheless, such an approach is seriously impaired by the unbalanced representation of the sequences from each compartment, most often favouring the host compartment [8, 17]. This bias can be minimised by specific library preparation (i.e. mRNA depletion), in silico normalisation procedures, and/or by investigating models in which the pathogen burden is high.

*Tenacibaculum maritimum* is a fish pathogen with a worldwide distribution, known for its lethal effects on a broad range of wild and farmed marine fish populations. Major efforts have been undertaken to lessen the impact of this pathogen and/or increase fish immune resistance [18]. The mechanisms of infection and fish response remain largely unknown which has significantly held back aquaculture development. Nevertheless, recent sequencing of *T. maritimum* strain NCIMB 2154^T^ genome has provided unprecedented information on the putative molecular mechanisms involved in virulence [19]. These authors note, for instance, that *T. maritimum* displays a large array of evolutionarily conserved stress resistance-related effectors, as well as an expanded capacity for iron mobilisation [1, 9].

Mucosal surfaces, especially skin mucus, are considered as the first barrier against pathogens [20]. This physical and chemical barrier constituted by mucus also includes host effectors for adaptive and innate immune response that orchestrate a complex interaction network with the commensal bacterial community [21, 22]. Recent studies on zebrafish (*Danio rerio*) raised in axenic conditions or in the presence of probiotic bacteria underlined the crucial role of the microbiota in the development of the immune system, mucosal homeostasis and resistance to stress and pathogens ([23], for review see [24]). Similarly, dysbiosis (i.e., the imbalance or alteration of the microbial ecosystem leading to a diseased status) is directly involved in the severity of a disease [25–27]. In French Polynesia, recurrent tenacibaculosis infections have been the main obstacle to sustainable local fish aquaculture. Indeed, *Tenacibaculum maritimum* affects the only locally-farmed orbicular batfish (*Platax orbicularis*) leading to very high mortality rates shortly after transferring hatchery fingerlings to off-shore marine cages. *T. maritimum* adheres and rapidly colonises mucosal surfaces [16, 28]. Infected fish show multiplication of *T. maritimum* on their external tissues leading to severe skin lesions followed by rapid fish death [16]. Nevertheless, the mechanisms by which *T. maritimum* can colonise and dominate skin microbiome are poorly known.

Here we combined dual RNAseq and 16S rRNA metabarcoding sequencing approaches to investigate the molecular responses of the host and microbiota (gene expression and microbiome taxonomic composition) simultaneously during *T. maritimum* infection and recovery phases, using orbicular batfish as a model. We also highlight putative virulence-related genes, based on comparisons of *T. maritimum* transcriptomic landscape during infection compared to in vitro cultures and explored genomic and genetic bases of resistance in *P. orbicularis*.

## Methods

### Animal husbandry

Fish were obtained from a mass spawning of six females and eight males induced by desalinisation. Broodstock included wild individuals caught in French Polynesia that had been maintained at the *Centre Ifremer du Pacifique* (CIP) hatchery facility for 7 years, under the supervision of the *Direction des Ressources Marines*. Details of eggs to fingerlings maintenance prior to the bacterial challenge are available in supplementary methods. Fingerlings were fed on commercial micro pellets ranging from 0.3 to 1 mm for the Micro-Gemma and Gemma ranges (Skretting, Stavanger, Norway) and from 1 to 1.3 mm for Ridley (Le Gouessant, Lamballe, France) according to the standard previously established [18]. Seawater supplied to both systems was pumped from the lagoon, filtered through a 300-μm sand filter and two 25- and 10-μm mesh filters and UV treated (300 mJ/cm^2^). The recirculating system included a 500-L biological filter to regulate levels of ammonia and nitrite. All tanks were supplied with saltwater held at 28.4 ± 0.3 °C at a constant photoperiod (12 L:12D) and oxygen saturation was maintained above 60% in the tanks with air distributed via air stones. Water renewal ranged from 36 to 360 L/h and new water input into the recirculating system was of 11 ± 1%. Levels of ammonia and nitrite were monitored once a week by spectrophotometry (HANNA Instruments®) to assess biofilter performance. Temperature, salinity and dissolved oxygen were measured daily (YSI®) and uneaten food and faecal material was removed once a day.

### Bacterial challenge and animal sampling

We used strain TF4 for the experimental infection. TFA4 was isolated from the skin of an infected *Platax orbicularis* in French Polynesia in 2013 and was shown to belong to *Tenacibaculum maritimum* by whole-genome sequencing, displaying an average nucleotide identity of 99.6% with the reference strain NCIMB 2154^T^ [29]. Strain TFA4 was cultivated in nutrient Zobell medium (4 g L^− 1^ peptone and 1 g L^− 1^ yeast extract Becton, Dickinson and Company, Sparks, MD in filtered and UV-treated seawater) under constant agitation (200 rpm) at 27 °C for 48 h.

On the infection day fish were 58 days post-hatching (dph) with an average weight 7.21 g ± 0.28 se). At this stage, a subsample of fish was transferred into 40-L tanks supplied with air and infected by the addition of 10 mL of a bacterial suspension of strain TFA4 to the tank water. Final bacterial concentration in the 40-L tanks, determined by the ‘plate-counting’ method, reached 4.10^4^ CFU.mL^− 1^. After 2 h of bathing, the fish were caught with a net, rinsed successively in two 40-L buckets filled with clean filtered UV-treated seawater and were transferred into three replicate tanks (50 animals / tank), hereafter called “infected” group. In parallel, other individuals were transferred into one 40-L tank where we added 10 mL Zobell medium to form a mock-treated group, hereafter called control. After 2 h of bathing, the fish were caught with a net, rinsed successively in two 40-L buckets filled with clean filtered UV-treated seawater and were transferred into two replicate tanks (50 animals / tank), hereafter called “control” group.

Twice a day, one third of the water in the tanks was replaced with filtered UV-treated seawater to maintain good water quality. This also made it possible to inactivate the *T. maritimum* in the sewage by bleach treatment. Dead animals were also collected and recorded at these times.

**Fig. 1:**
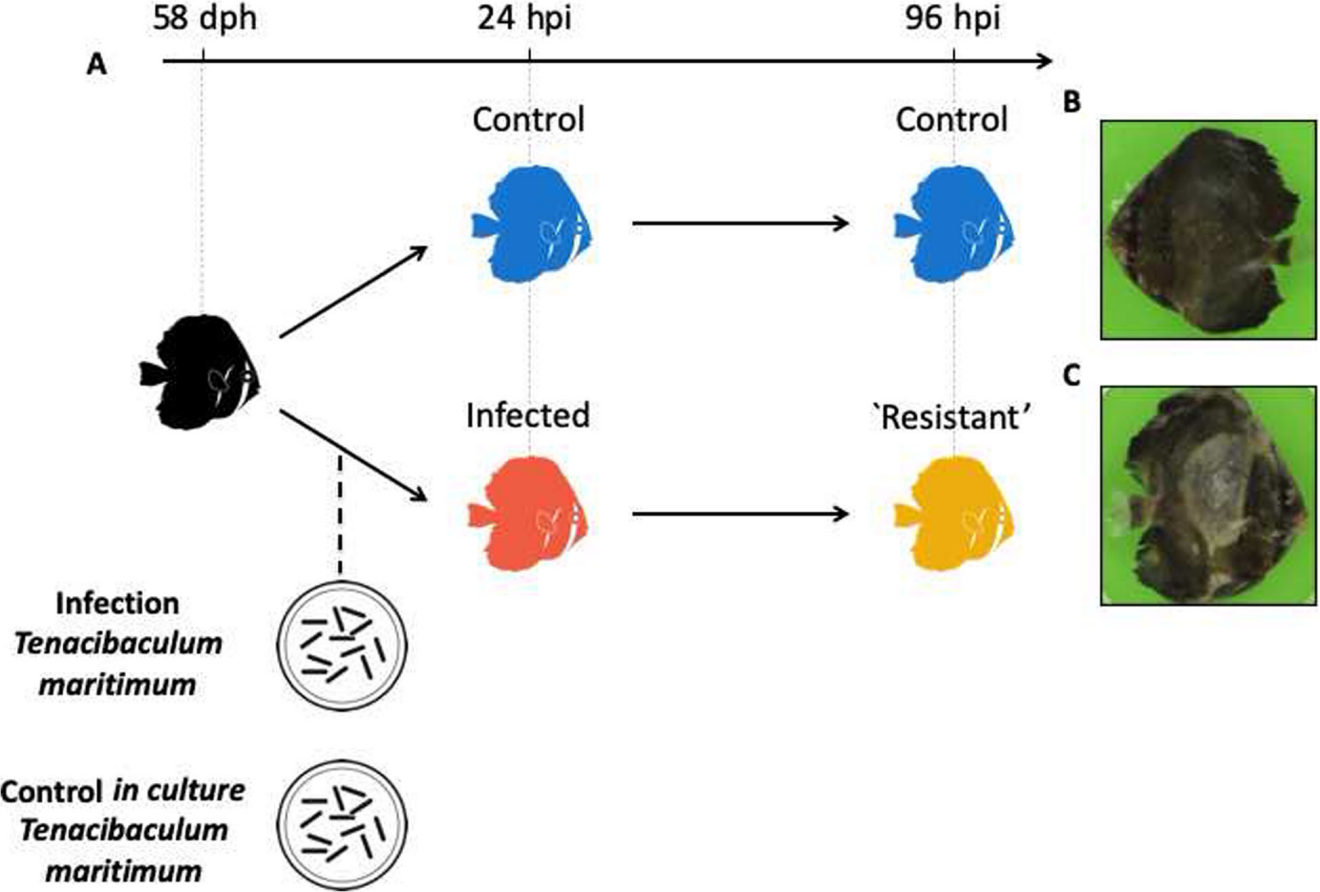
Experimental design and individual photographs. **a** Experimental infection was conducted at 58 dph, after random sampling of five individuals per tank to assess initial weight. At 24 and 96 h hpi, five individuals per tank (*N* = 15 individuals, *infected*_*24h*_; *N* = 15 individuals, *resistant*_*96h*_; *N* = 10 individuals, *control*_*24h*_ and *N* = 10 individuals, *control*_*96h*_) were sampled using swabs. The same individuals served for host and microbiome transcriptomics and for microbiome metabarcoding. **b** Photograph of a control fish (control_*24h*_); **c** Photograph of an infected fish (infected_*24h*_) showing typical skin lesions associated with tenacibaculosis

Samplings consisted of five individuals per tank at 24 hpi and 96 hpi (Fig. 1a). Our design consisted of four groups, namely *control*_*24h*_ (*N* = 10 individuals)_*,*_ *control*_*96h*_ (*N* = 10 individuals), *infected*_*24h*_ (*N* = 15 individuals) and *resistant*_*96h*_ (*N* = 15 individuals)_._ For each sampling, at 24 hpi and 96 hpi, individuals were lethally anaesthetised using a benzocaine bath (150 mg. L^− 1^) and a lateral photograph was taken using a digital fixed camera (Leica Microsystems; Fig. 1b and c). Microbiome and host sampling consisted in making gentle fish skin smears with sterile swabs that were directly placed in TRIZOL Reagent (Life Technologies) on ice to prevent RNA degradation. Swabs were disrupted using a mixer mill MM200 (Retsch) for 5 min at a frequency of 30 Hz and stocked at − 80 °C for later analysis. In parallel, water was also sampled from each tank but was not included in the analysis due to its very low DNA yield. At 115 h post-infection (hpi), all living infected animals were considered as resistant and the challenge was ended. All the remaining fish were euthanised.

### *T. maritimum* in vitro liquid culture sampling

The TFA4 strain was cultivated in 6 mL Zobell medium under constant agitation (200 rpm) at 27 °C for 48 h, following exactly the same procedure and time of incubation as the culture used for bacterial challenge. Five culture replicates were performed. For each replicate, 4 mL at 10^8^ CFU/mL were centrifuged 5 min at 10,000 g at room temperature. Three inox beads and 2 mL TRIZOL (Life technologies) were quickly added to each bacterial pellet and cells were immediately disrupted using a mixer mill MM200 (Retsch) for 5 min at 30 Hz to prevent RNA degradation. RNA was extracted following manufacturer’s instructions, using a high salt precipitation procedure (0.8 M sodium citrate and 1.2 M NaCl per 1 mL of TRIZOL reagent used for the homogenisation) to reduce proteoglycan and polysaccharide contamination. Quantity, integrity and purity of total RNA were validated by both NanoDrop readings (NanoDrop Technologies Inc.) and on a Bioanalyzer 2100 system (Agilent Technologies). DNA contaminants were removed using a DNAse RNase-free kit (Ambion). A total of five RNA samples (1.048 ± 0.019 μg) were further dried in RNA-stable solution (Thermo Fisher Scientific) following manufacturer’s recommendations and shipped at room temperature to McGill sequencing platform services (Montreal, Canada). One library was removed prior to sequencing because it did not meet the minimum quality requirements.

### Fish mortality and cortisol measurements

Mortality was recorded at 0, 19, 24, 43, 48, 67, 72, 91, 96 and 115 hpi. To estimate log-rank values, we used the non-parametric Kaplan-Meier approach implemented in the *survival* R package [30]. Differences in survival probability were considered significant when *P* < 0.05. We assessed stress levels in fish by measuring scale cortisol content [31]. The scales were collected from both sides of each individual, washed and then vortexed three times (2.5 min; 96% isopropanol) to remove external cortisol origination from the mucus. Residual solvent traces were evaporated under nitrogen flux and samples frozen at − 80 °C. To ensure the scales were dry, they were lyophilised for 12 h and then ground to a powder using a ball mill (MM400, Retsch GmbH, Germany). Cortisol content was extracted from ~ 50 mg of dry scale powder by incubation in 1.5 mL methanol (MeOH) on a 30 °C rocking shaker for 18 h. After centrifugation at 9500 g for 10 min, the supernatant was evaporated using a rotary evaporator and reconstituted with 0.2 mL of EIA buffer from a Cortisol assay kit (Neogen® Corporation Europe, Ayr, UK). Cortisol concentrations were determined in 50 μL of extracted cortisol using a competitive EIA kit (Neogen® Corporation Europe, Ayr, UK) according to a previously published protocol [32]. After validation of normality and homoscedasticity, differences among groups were tested using a two-way ANOVA followed by Tukey’s HSD post-hoc tests. Differences were considered significant when *P* < 0.05.

### RNA and DNA extraction and sequencing

#### Dual RNAseq

Total RNA was extracted using the same procedure as described above. RNA was then dried in RNA-stable solution (Thermo Fisher Scientific) following manufacturer’s recommendations and shipped at room temperature to McGill sequencing platform services (Montreal, Canada). Ribo-Zero rRNA removal kit (Illumina, San 260 Diego, Ca, USA) was used to prepare rRNA-depleted mRNA libraries that were multiplexed (13–14 samples per lane) and sequenced on a HiSeq4000 100-bp paired-end (PE) sequencing device. Infected individuals 24 hpi were sequenced twice to insure sufficient coverage (Table S1).

#### Short-read 16S rRNA MiSeq microbiome sequencing

Total DNA was extracted from the same TRIZOL Reagent (Life Technologies) mix described above. DNA quantity/integrity and purity were validated using both a Nanodrop (NanoDrop Technologies Inc.) and a Bioanalyzer 2100 (Agilent Technologies). The V4 region was amplified by PCR using modified 515F/806rb primer constructs (515F: 5′-GTGYCAGCMGCCGCGGTAA-3′; 806rb: 5′- GGACTACNVGGGTWTCTAAT-3′) recommended for microbial survey [33]. Amplicon libraries were multiplexed and sequenced on a single lane of a MiSeq 250 bp PE Illumina machine at Genome Québec McGill, Canada. Details of the sequencing statistics are given in Table S2.

#### Full 16S rRNA Nanopore sequencing

For a broad range amplification of the 16S rRNA gene, DNA was amplified using the 27F/1492R barcoded primer products (27F: 5′- AGAGTTTGATCMTGGCTCAG-3′; 1492R: 5′- TACGGYTACCTTGTTACGACTT-3′). In the PCR experiment, we included eight randomly selected individuals from the *infected*_*24h*_ group, two negative PCR controls (clean water) and one positive control (Acinetobacter DNA).

The PCR mixtures (25 μL final volume) contained 10 ng of total DNA template or 10 μL of water, with 0.4 μM final concentration of each primer, 3% DMSO and 1X Phusion Master Mix (Thermo Fisher Scientific, Waltham, MA, USA). PCR amplifications (98 °C for 2 min; 30 cycles of 30 s at 98 °C, 30 s at 55 °C, 1 min at 72 °C; and 72 °C for 10 min) of all samples were carried out in triplicate in order to smooth out intra-sample variance. Triplicates of PCR products were pooled and purified by 1x AMPure XP beads (Beckmann Coulter Genomics) clean-up. Amplicon lengths were measured on an Agilent Bioanalyzer using the DNA High Sensitivity LabChip kit, then quantified with a Qubit Fluorometer.

An equimolar pool of purified PCR products (except for negative controls) was made and one sequencing library was finally prepared from 100 ng of the pool using the 1D Native barcoding genomic DNA protocol (with EXP-NBD103 and SQK-LSK108) for R7.9 flow cells run (FLO-MAP 107) then sequenced on a MinION device. Details of the sequencing statistics are provided in the Supplementary Material.

### Microbiota analyses

#### Microbiome dynamics with the MiSeq short-reads dataset

Quality of the raw reads and presence of adaptors was determined using fastQC v0.11.9 (https://www.bioinformatics.babraham.ac.uk/projects/fastqc/). The remaining adaptor sequences were removed using BBDuk software (last modified January 25, 2018), implemented in BBtools package (https://jgi.doe.gov/data-and-tools/bbtools/), forcing left trimming of 20 bp *‘forcetrimleft = 20’.* We followed DADA2 [34] standard procedure for detecting presence of remaining adaptors, reads filtering, error-correction and for building the exact sequence variant (ESV) table. Briefly, reads were filtered using default parameters (maxN = 0, maxEE = c (2,2), truncQ = 2) and trimmed at 190 bp. Errors modelling and correction was conducted using the pooled option on the merged reads. Putative chimeras were detected de novo and removed. Finally, taxonomy was assigned using the formatted NCBI RefSeq 16S rRNA database supplemented by RDP (https://benjjneb.github.io/dada2/training.html) which was complemented with the species-assignments training dataset to improve species levels assignment. Dataset was imported to QIIME2 platform v2019.10 to build the phylogenetic trees. We explored alpha-diversity (Shannon, Fisher indexes) and beta-diversity (Bray-Curtis, unweighted and weighted Unifrac distances) using the *phyloseq* R package [35]. Dissimilarity between samples was assessed by principal coordinates analysis (PCoA). Differences in alpha-diversity were tested using pairwise Wilcoxon rank tests and were considered significant when Holm adj. *P* < 0.01. Differences in beta-diversity were tested using Anosim (1000 permutations) as implemented in the ‘*anosim’* function of the *vegan* R package [36]. Differences were considered significant when *P* < 0.01. We explored pairwise differences using the *‘pairwise.adonis’* function of the pairwise.adonis R package (https://github.com/bwemheu/pairwise.adonis). Differences were considered significant when BH adj. *P* < 0.01. We also searched for a ‘core’ microbiome of fish skin, considering those ESVs present in all the individuals across all treatments (infected, control and resistant) as belonging to this group. We finally searched for significant differences in specific ESV abundance across groups using Wald tests implemented in the *DESeq2* R package [37]. We used the *apeglm* method for log2FC shrinkage to account for dispersion and variation of effect size across individuals and treatments, respectively [38]. Differences were considered significant when FDR < 0.01 and |FC| > 2.

#### Microbiota consensus representation using Nanopore dataset

Sequences were called during the MinION run with MinKnow software (v. 1.7.14). The demultiplexing and adaptor trimming were done with the porechop tool (https://github.com/rrwick/Porechop) using the option discard_middle. For each barcode, all Nanopore reads were mapped on the GreenGenes database (v.13.5, http://greengenes.lbl.gov) with minimap2 (v2.0-r191) with the ‘map-ont’ pre-set options [39]. All reference sequences of the GreenGenes database covered by more than 0.01% of all reads were kept for the next step. A second round of mapping (using the same parameters) was done on the selected references in order to aggregate reads potentially mis-assigned during the first round of mapping. SAMtools and BCFtools were used to reconstruct consensus sequences for each reference sequence covered by more than 10 Nanopore reads with the following programs and options: mpileup -B -a -Q 0 –u; bcftools call -c --ploidy 1; vcfutils.pl vcf2fastq. Individuals and consensus sequences were blasted (e-value < 10^− 5^) against the NCBI nt database (https://ftp.ncbi.nlm.nih.gov/blast/db; accessed October 2019).

### Compartment-specific differential expression analyses

#### Read pre-processing

For each individual, raw reads were filtered using Trimmomatic v0.36 [40], with minimum length 60 bp, trailing 20 and leading 20. Filtered PE reads were mapped against a combined reference including the host’s transcriptome (See Supplementary Material Tables S1 and S3 for details of the transcriptome assembly) and the genomes of *Alteromonas mediterranea* strain: AltDE1 (Genbank accession: GCA_000310085.1), and *Pseudoalteromonas phenolica* strain: KCTC 12086 (Genbank accession: GCA_001444405.1), *Tenacibaculum maritimum* strain: NCIMB 2154 T (Genbank accession: GCA_900119795.1), *Sphingobium yanoikuyae* strain ATCC 51230 (Genbank accession: GCA_000315525.1), *Vibrio alginolyticus* strain: ATCC 17749 (Genbank accession: GCA_000354175.2,) and *Vibrio harveyi* strain: ATCC 43516 (Genbank accession: GCA_001558435.2). To prevent multi-mapping biases, we used GSNAP v2017-03-17 [41] with minimum coverage set at 0.9, a maximum of five mismatches allowed, and removal of improperly paired and non-uniquely mapped reads (option ‘concordant_uniq’). Reads with low mapping quality (MAPQ) were removed using SAMtools v1.4.1 [42] with the minimum MAPQ threshold fixed at five. A matrix of raw counts was built using HTSeq-count v0.9.1 [43]. Transcripts from the host and bacterial species origin were then separated into different contingency tables using homemade scripts.

#### Host transcriptome analysis

Low coverage transcripts with count per million (CPM) < 1 in at least nine individuals were removed, resulting in a total of 22,390 transcripts. Similarly, transcript over-representation was assessed using *‘majSequences.R’* implemented in the SARTools suite [44]. We used distance-based redundant discriminant analysis (db-RDA) to document genetic variation among groups and correlation with group (infected or control), weight and time (24 and 96 hpi) as the explanatory variables. Briefly, we computed Euclidean distances and PCoA using the ‘*daisy*’ and ‘*pcoa*’ functions, respectively, implemented in the *ape* R package [45]. PCo factors (*n* = 6) were selected based on a broken-stick approach [46, 47] and used to produce a db-RDA. Partial db-RDAs were used to assess the factor effect, checking for the other factor variables. We tested the significance of the models and individual factors using 999 permutations. Effects were considered significant when *P* < 0.01.

Differential expression was assessed using the *DESeq2* R package [37], using pairwise comparisons with Wald tests. Logarithmic fold changes (logFC) were shrunk using the ‘*apeglm*’ method, implemented in the *DESeq2* R package [37], to account for dispersion and effect size across individuals and treatments [38]. Differences were considered significant when FDR < 0.01 and FC > 2. Group comparisons included *infected*_*24h*_ vs *control*_*24h*_ and *resistant*_*96h*_ vs *control*_*96h*_. Gene ontology (GO) enrichment was tested using GOAtools v0.6.5 [48] and the go-basic.obo database (release 2017-04-14) using Fisher’s test. Our background list included the ensemble of genes in the host transcriptome. Only GO terms with Bonferroni adjusted *P* < 0.01 and including at least three differentially expressed genes were considered. Significant GO enriched terms were used for semantic similarity-based clustering in REVIGO (http://revigo.irb.hr/).

#### *Tenacibaculum maritimum* gene expression in vitro or during infection

A validation step for searching for transcript over-representation was assessed using *‘majSequences.R’* implemented in the SARTools suite [44], similarly to the fish transcriptome. Most represented sequences were attributed to *ssrA*-coding genes, but represented less than 8% of the total library. We applied a similar shrinkage method and pairwise comparisons (infected vs in vitro), as for the host comparisons, but used more stringent thresholds as commonly observed in similar studies [8], and considered significant differences when FDR < 0.01 and FC > 4. Gene ontology (GO) enrichment was similar to the methods used for the host.

### Species-specific weighted co-network gene expression analyses in the host

We built a signed weighted co-expression networks for the host compartment to cluster co-expressed genes and identify putative driver genes using the *WGCNA* R package [49]. Variation in normalised counts were previously controlled using the ‘*vst*’ method implemented in the *DESeq2* R package [37].

We reduced the expression noise in the dataset by keeping only transcripts with minimum overall variance (> 5%). Briefly, we fixed a soft threshold power of 14 using the scale-free topology criterion to reach a model fit (|R|) of 0.80. The modules were defined using the ‘*cutreeDynamic*’ function (minimum 30 genes by module and default cutting-height = 0.99) based on the topological overlap matrix, a module eigengene distance threshold of 0.25 was used to merge highly similar modules. For each module, we defined the criteria for module membership (kME, correlation between module eigengene value and gene expression). We looked for significant correlation (Pearson’s correlation; *P* < 0.001) between modules and physiological data, including cortisol levels (pg.mg^− 1^ in scales), fish weight (g) and treatment (coded ‘1’ for *control*_*24h*_, *control*_*96h*_ and *resistant*_*96h*_ and ‘2’ for *infected*_*24*_). Gene ontology (GO) enrichment for each module was tested using same protocol and parameters as described above.

### The genetic bases of fish resistance

We further explored the putative genetic variation between resistant and infected fish by focusing on resistant fish because of their established phenotype, (i.e. survivors with no signs of lesions after bacterial challenge). We followed GATK recommendations for SNP identification based on RNAseq data. Briefly, BAM files were pre-treated using the ‘*CleanSam*’ function, duplicates were picked out with the ‘*MarkDuplicates*’ function, and cigar string split with the ‘*SplitNCigarReads*’ function. All functions were implemented in GATK v4.0.3.0 software [50, 51]. Final SNP calling was conducted with Freebayes v1.1.0 (https://github.com/ekg/freebayes) requiring minimum coverage of 15 and minimum mapping quality of 20, forcing ploidy at 2 and removing indels (‘*—no-indels’*) and complex polymorphisms (‘*—no-complex’*). The raw VCF file was filtered for minimum allele frequency (‘*—min_maf = 0.2*’), minimum coverage (‘*—minDP = 20’*) and using Vcftools v0.1.14 [52] to allow no missing data. We computed relatedness (‘*—relatedness2’*) within and among groups with Vcftools v0.1.14 [52]. We further used distance-based redundant discriminant analysis (db-RDA) to document genetic variation among groups and correlation, with cortisol, treatment and weight as the explanatory variables. Briefly, we computed Euclidean distances and PcoA using the ‘*daisy*’ and ‘*pcoa*’ functions, respectively, implemented in the *ape* R package [45]. Pco factors (*n* = 6) were selected based on a broken-stick approach [46, 47] and used to produce a db-RDA. We tested the model significance using 999 permutations, effects were considered significant when *P* < 0.01.

## Results

### Fish weight, cortisol levels and mortality

**Fig. 2:**
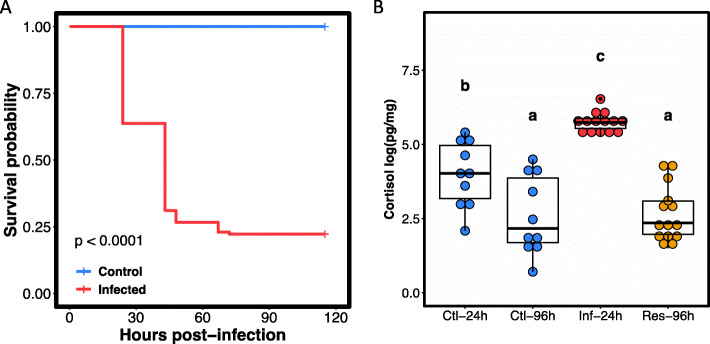
Kaplan–Meier survival estimates and fish scale cortisol levels. **a** Kaplan–Meier survival curves for control (blue) and infected (red) groups over the 115 hpi of the experiment. Values represent the probability of survival (0 to 1). Survival was checked at 0, 19, 24, 43, 48, 67, 72, 91, 96 and 115 hpi. **b** Scale cortisol levels are expressed on a logarithmic (Log10) scale. Ctl-24 h: *control*_*24h*_; Ctl-96 h: *control*_*96h*_, Inf-24 h: *infected*_*24h*_; Res-96 h: *resistant*_*96h*_, groups. Different letters indicate significant differences, *P* < 0.05, Tukey’s HSD test

Mortality rate in challenged fish reached 77.36 ± 18.35 (mean ± standard error; se) while no mortality was observed in the control group (Kaplan-Meier analysis, *P* < 0.001; Fig. 2a). Survival probability strongly decreases in the first 72hpi (starting at 24hpi) in the infected group. Cortisol levels in fish scales vary significantly across groups (ANOVA; *F* = 9.46; *P* < 0.01; Fig. 2b). Overall, cortisol levels were higher in the *infected*_*24h*_ group compared with all the other groups (Tukey’s HSD; *P* = 0.01). Cortisol levels in the *control*_*24h*_ group were also higher than in the *control*_*96h*_ and *resistant*_*96h*_ groups (Tukey’s HSD; *t* = − 3.28; *P* = 0.01 and *t* = − 3.42; *P* < 0.01, respectively). However, no difference was observed between *control*_*96h*_ and *resistant*_*96h*_ groups (Tukey’s HSD; *t* = 0.12; *P* = 0.99).

### Dynamics of host transcriptomic response to infection and search for genomic bases of resistance

Mean number of paired-end raw reads reached 65.44 M ± 23.1 sd and 25.46 ± 4.31 sd, for *infected24h* and for *control24h, control96h and resistant96h*, respectively. Global mean unique mapping rate for skin smear samples reached 71.64 ± 2.99% relative to a combined reference for host and microbe compartments. Datasets were predominantly composed of host-origin sequences (mean 86.57 ± 13.48%), with the *infected*_*24h*_ group showing a significantly higher proportion of reads of non-host origin [mean 30.70% ± 0.03 se] than other groups (Dunn’s test; Benjamini-Hochberg adj. *P* < 0.05; Figure S1). Details of the host transcriptome and individual mapping are provided in Tables S1 and S3.

#### Fish response to infection

Differential expression analyses revealed strong differences in host gene expression profiles between *control*_*24h*_ and *infected*_*24h*_, with a total of 3631 and 2388 down- and up-regulated genes in *infected*_*24h*_, respectively, compared with *control*_*24h*_, (|FC| > 2; FDR < 0.01; Table S4). The *infected*_*24h*_ group responded to infection mainly by activating immune system response (Biological Process; BP), sterol biosynthetic process (BP), defence response (BP), inflammatory response (BP), regulation of biological quality (BP), lipid metabolic process (BP), iron ion homeostasis (BP), complement binding (Molecular Function; MF), heme binding (MF), oxidoreductase activity (MF), sulfur compound binding (MF), and (1- > 3)-beta-3-D-glucan binding (MF). A complete list of GO enrichment for each module is provided in Table S4.

**Fig. 3:**
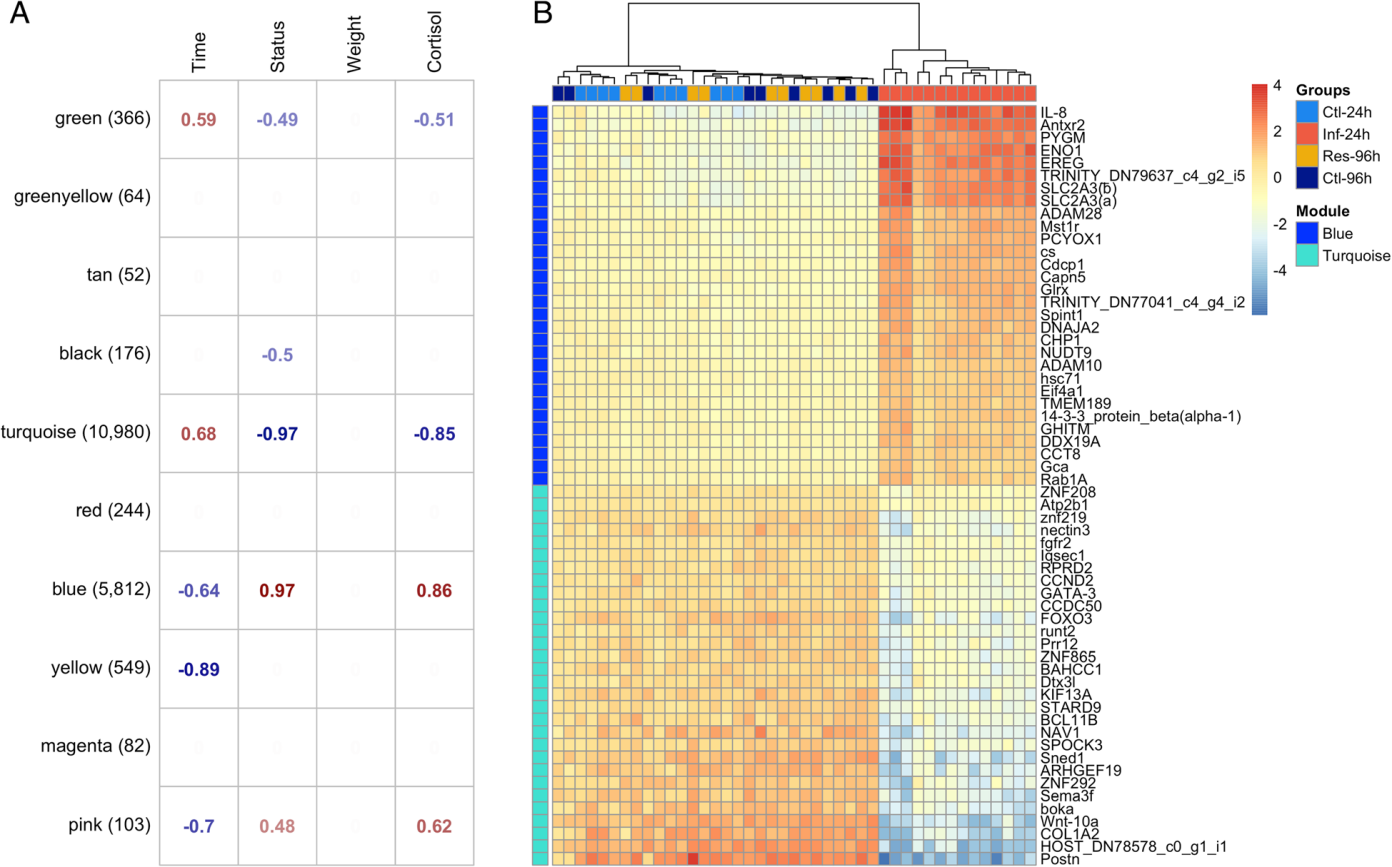
Signed co-expression network analysis for *P. orbicularis*. **a** Correlation matrix for *P. orbicularis.* Values in the cells represent significant (*P* < 0.01) Pearson’s correlation of module eigenvalue to physiological parameters (top panel). Names (left panel) are arbitrary color-coded names for each module; values in parenthesis represent the number of genes per module. Empty cells indicate non-significant correlations (*P* ≥ 0.01). Individual cortisol [log (pg.mg^− **1**^)] and weight (g) are continuous values. Time (24 hpi and 96 hpi) and Status (coded 1 for *control*_*24h*,_ *control*_*96h*_ and *resistant*_*96h*_ groups and 2 for i*nfected*_*24h*_) are discrete numeric values. b Heatmaps of the top 30 genes in module_blue-host_ and module_turquoise-host._ Scales represent Log2 (prior 2) of the individual expression levels. Individuals were clustered using hierarchical clustering procedures implemented in the *pheatmap* R package [53]

We then used co-expression network analysis (WGCNA) to draw clusters of co-regulated genes associated with discrete (treatment) or continuous variables (weight and cortisol) and to identify putative hub genes. No gene module correlated significantly with fish mass, suggesting that mass had no significant effect on gene expression profiles. A total of three modules showed negative correlations (*P* < 0.01) with disease status (coded 1 for *control*_*24h*,_ *control*_*96h*_ and *resistant*_*96h*_ groups and 2 for *infected*_*24h*_), namely module_turquoise-host_ (*r* = − 0.97, *P* < 0.001), module_black-host_ (*r* = − 0.5, *P* < 0.001) and module_green-host_ (*r* = − 0.49, *P* < 0.001; Fig. 3). Inversely, two modules showed positive correlation with the treatment, namely module_blue-host_ (*r* = 0.97, *P* < 0.001) and module_pink-host_ (*r* = 0.48; *P* = 0.001). Almost all of these modules (with the exception of module_black-host_) also correlated significantly with cortisol levels. The genes found up-regulated in *control*_*24h*_ clustered mostly in module_turquoise-host_ (*n* = 3468; 95.5%), module_black-host_ (*n* = 72; 2.0%) and module_green-host_ (*n* = 39; 1.0%). Nearly all the genes found to be up-regulated in *infected*_*24h*_ clustered in module_blue-host_ (*n* = 2352; 98.5%). Not surprisingly, the main driver genes (‘hub-genes’) include several transcriptional activators, such as, for module_turquoise-host_, several Zinc finger proteins, Transcription factor GATA-3 (*gata-3*), Forkhead box protein O3 (*foxpo3*), activators of the autophagy pathways and the main drivers of naïve specific T-cell differentiation and activation [54], Runt-related transcription factor 2- (*runt2*) coding genes, involved in osteoblast differentiation, mineral- depositing cells and sialoproteins [55, 56]. In module_blue-host_, ‘hub-genes’ mainly reported actors of the innate immune system, inflammatory response, wound healing, oxidative and adhesion activity (Fig. 3b).

#### Genomic bases of resistance

We found 38 differentially expressed genes (DEGs) between *control*_*96h*_ and *resistant*_*96h*_ (16 and 22 down and up-regulated in *resistant*_*96h*_, respectively; |FC| > 2; FDR < 0.01). GO analyses show that adaptive immune response (BP) tends to be activated (uncorrected *P* < 0.001) in *resistant*_*96h*_ that includes genes related to pathogen recognition and immune response, such as C-type lectin domain family 4 member M, low affinity immunoglobulin gamma Fc receptor II-like and T-cell receptor beta variable 7-2-coding genes. Inversely, *resistant*_*96h*_ show inactivation of ERK1 and ERK2 cascade (BP) regulation and repressors of the response to wounding (BP), regulation of the transforming growth factor-beta secretion (BP), alcohol biosynthetic process (BP) and regulation of interleukin-8 production (BP). However, GO enrichments were not considered significant under our threshold (Bonferroni adj. *P >* 0.05). A total of 27 (71.1%) out the 38 DEGs identified also showed different expression levels between *control*_*24h*_ and *infected*_*24h*_. Among the 11 remaining genes (28.9%), we found Arf-GAP with dual PH domain-containing protein 1, C-type lectin domain family 4 member M, protein KIAA1324-like homolog, Ankyrin repeat and fibronectin type-III domain-containing protein 1 and T-cell receptor beta variable 7-2, up-regulated in *resistant*_*96h*_ group. Inversely, we found the Sal-like protein 1, Early growth response protein 1 and the Low affinity immunoglobulin gamma Fc receptor II-like down-regulated in *resistant*_*96h*_. The complete list of DEGs and GO term enrichment is provided in Table S4.

Finally, we searched for genetic variation (SNPs) across *resistant*_*96h*_ and *infected*_*24h*_ (two established phenotypes) in order to identify putative variants associated with resistance capacities. We identified a subset of 13,448 filtered bi-allelic SNPs. Genetic variation analyses did not suggest any significant difference among groups (relatedness, Fst) and was not correlated with cortisol levels or fish mass of any of the groups based on the 13,448 markers (PERMANOVA; 1000 permutations; *P* = 0.18; Figure S2).

### Microbiome flexibility and interactions among pathogen species and host response

#### Dynamics of microbiota communities on fish skin

The MiSeq sequencing strategy with amplification of the 16S rRNA V4 region of the 16S rRNA gene resulted a mean number of PE of 228,929.857 ± 39,633.05 sd (Table S2). One individual in the *control*_*96h*_ condition was removed due to low sequencing yield. Species richness (Shannon) was lower in *infected*_*24h*_ than in other groups (Mann-Whitney-Wilcoxon; MWW; Holm adj. *P* < 0.001). Similarly, *control*_*24h*_ showed reduced species diversity values compared with *control*_*96h*_ (MWW; Holm adj. *P* < 0.001), but no difference was observed between *resistant*_*96h*_ and *control*_*96h*_ (MWW; Holm adj. *P* = 0.26; Figure S3A). Similar results were found with Fisher’s indices (Figure S3B). Bacterial communities varied significantly across groups (Anosim; *R* = 0.62; *P* < 0.001; 1000 permutations; weighted UniFrac distances), with all groups being different from each other (pairwise.adonis; R2 = [0.22 – 0.75]; BH adj. *P* = [0.001 – 0.004], wUniFrac). The variation was consistent for all the beta-diversity distances / indexes tested (UniFrac, wUniFrac and Bray-Curtis; partial Mantel test; *r* = 0.52; *P* = 0.001). The ESVs associated with *Tenacibaculum (Flavobacteriales)* are largely enriched in *infected*_*24h*_ compared with *control*_*24h*_, but also significantly enriched in *resistant*_*96h*_ compared with *control*_*96h*_ (shrunken |log2FC| > 2; FDR < 0.01; Figure S4).

#### Long-read refinement of bacterial communities in infected fish

Results from Nanopore sequencing on full 16S rRNA sequences served to refine the taxonomy at the species levels, which might be limited with short-reads approaches. We amplified the full 16S rRNA of eight individuals randomly subsampled from the 24 hpi infected group, resulting in a mean number of SE reads of 60,019.62 ± 33,778.99 sd after pre-processing (the individual with the lowest coverage had a total of 29,520 sequences). Nanopore shows an over-dominance of *T. maritimum* in *infected*_*24*_ (73.51 ± 4.89%) and confirmed the presence of other genera, including *Vibrio*, *Polibacter*, *Alteromonas* and *Pseudoalteromonas* (Figure S5). Among these genera, some species, such as *V. harveyi* [57], are known as potential fish pathogens, while others (*Pseudoalteromonas*) have been proposed as putative probiotics [58].

### Microbial compartment transcriptomic activity

#### Gene expression of *Tenacibaculum maritimum* during experimental infection vs in vitro

**Fig. 4:**
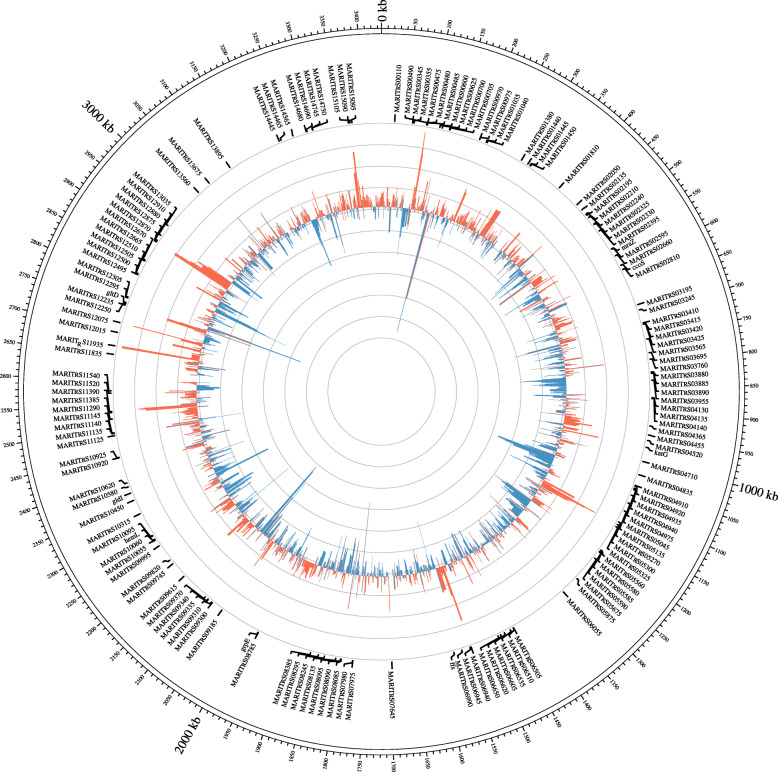
Circos plot of in vitro and in vivo expression comparisons in *T. maritimum*. External line represents mean shrunken log2FC in vitro (negative values) compared with in vivo (positive values). Names of the genes differentially expressed are reported on the outer layer. Circos positions were based on *T. maritimum* NCIMB 2154^T^ genome information. Positions indicated by external ticks are reported in millions of bp

We examined the gene expression levels for *T. maritimum* in the fish during the peak of infection compared with in vitro to highlight putative genes associated with virulence (Fig. 4). Mean total mapped reads against *T. maritimum* in vivo reached 5.65 M ± 0.82 se (Figure S6), which is sufficient to conduct differential expression analysis [59].

We found a total of 72 and 142 DEGs up-regulated during experimental infection (in vivo) and in vitro, respectively (Shrunken |log2FC| > 2; FDR < 0.01). We only found the sulfate assimilation (BP) function enriched in vitro (Bonferroni adj. *P* < 0.1). Among the GO positively enriched during experimental infection (Bonferroni adj. *P* < 0.1) we found the glucan catabolic process (BP), external encapsulating structure part (cellular component, CC), pattern binding (MF) and the antibiotic catabolic process (BP). These processes include genes already highlighted in genome scale comparisons of *Tenacibaculum* species as possible virulence-associated factors, such as the catalase/peroxidase *katG*, cholesterol-dependent cytolysin collagenase, the pair SusC-SusD as well as all seven other genes found in the polysaccharide utilization loci (PUL) system [19] (Figure S7). This PUL encompass major virulence-related factors in *T. maritimum*, involved in the utilization of sialic acids from the host [19] (Figure S8). In parallel, we also detected putative candidates involved in host membrane interactions and integrity such as Ulilysin, Streptopain, Pneumolysin toxin and peptidoglycan-associated factor lipoprotein or adhesines_._ A complete list of GO is provided in Table S4.

## Discussion

Tenacibaculosis is a worldwide fish disease responsible for considerable farmed fish mortality events, but knowledge is lacking on the microbiome kinetics during infection and the concomitant host–pathogen interaction. The dual RNAseq method was chosen as it provides unparalleled simultaneous data on the molecular features of the infection. It is particularly suitable for systems characterised by a massive pathogen burden with readily accessible material but for which cultures are not available [17, 60]. Here, we adapted this approach for tenacibaculosis in *P. orbicularis* fish skin samples with the goal for comprehensively assessing the genomic basis and kinetics of infection as well as associated resistance mechanisms.

### Infection modulates innate and adaptive host immune effectors

Bath exposition of *T. maritimum* was highly efficient at inducing tenacibaculosis in juvenile orbicular batfish. The low survival rate and kinetics of infection support what is usually observed for other fish species [16, 28, 61]. Infected fish, sampled at the peak of infection (24 hpi), show large skin lesions characteristic of tenacibaculosis together with a high cortisol concentration in their scales [31]. Cortisol mediates changes in individual energy balance (e.g. mobilisation of energy stores, immunity, cognition, visual acuity or behaviour) [62, 63]. This initial cascade of physiological and behavioural changes enables the organism to cope with acute stressors by mobilising adequate bodily functions, while concurrently inhibiting non-essential functions (e.g. reproduction, digestion) [64]. Here, increasing cortisol level reflects a local stress response to an unfavourable environment and is most likely involved in triggering the fish rapid immune response [65].

As expected, fish immune response, especially the innate immune system is strongly solicited at 24 hpi. Infected fish show activation of acute inflammatory response, mainly through driver genes, including interleukin-8 (IL-8) [66], but also activation of pathogen recognition receptors (PRRs), chemokines and antimicrobial-related humoral effectors. For instance, infection triggers co-expression of the cascade Toll-like receptor 5 (TLR5) and Myeloid differentiation primary response protein (MyD88), as previously reported in bony fish during bacterial infection [67]. However, the diversity of fish immune actors combined with the relatively limited knowledge we have on specific effector functions significantly hampered the comprehensive understanding of the mechanisms involved in our non-model species. For instance, in parallel to the TLR5, several other TLRs show reduced expression in infected fish, including TLR2 type-1, TLR-8 and non-mammalian (‘fish-specific’) TLR21. Despite previous effort towards assessing diversity of TLR sequences, protein-specific function remains poorly known in teleosts [68]. Similar observations have been made for the complement system, specifically complement C3, a key component of the immune system involved in ‘complementing’ antibodies for bacterial cell killing [69], for which several isoforms are reported in the *Platax* transcriptome. The different isoforms here have divergent patterns of expression (both up- and down-regulated in the *infected*_*24h*_ group), which support previously observed differences in target surface binding specificities [70].

Innate immune response is generally tightly linked to cellular homeostasis regulation and precedes adaptive immune response. The ability of the fish to maintain cellular homeostasis during infection is of primary importance when facing infection, and mechanisms include redox, biological quality control (autophagy) as well as ion level maintenance [71, 72]; all of which were found to be affected in *Platax*. For instance, infected fish largely activate effectors of iron ion homeostasis. Iron, albeit largely present in the environment, is poorly accessible to organisms and iron sequestration and maintenance is a major mechanism developed by the host to limit pathogen growth as well as to regulate macrophage cytokine production [73]. In parallel, *infected*_*24*_ individuals activated the (1- > 3)-beta-D-glucan binding. β-glucans, similarly to pathogen-associated molecular patterns (PAMP), are recognized as “dangers” signals which trigger conserved mechanisms of immune response (through PRRs, C-lectin and/or TLRs) across vertebrates and invertebrates [74, 75]. Indeed, supplementation of β-glucan stimulates immune response in fish and increases resistance of the host to viruses and other pathogens (probably by reducing bacterial adhesion through lectin binding [76]); it therefore represents a promising immunostimulant for aquaculture [75, 77]. Effects of β-glucan vary depending on species, exposure time, source of glucan, organs and markers monitored [74, 78] and further studies will be needed to evaluate its potential at the production scale.

The adaptive immune response was also modulated at 24 hpi and its fine-tuned orchestration offers the opportunity to separate the preferential immune paths that can fight against *T. maritimum* infection. We identified several hallmarks of differentiated T-cells, indicative of the specialisation of the adaptive immune response to *T. maritimum* infection. Among the main driver genes of the response to infection in *Platax*, we noted a reduced expression of *foxp3* and *gata-3* in *infected*_*24h*_. Both transcription factors are important regulators of the fate of Naïve CD4+ naïve T-cells, encouraging differentiation to T-regulatory (Treg) [79] and T-helper 2 (Th2) cells [54], respectively. Similarly, we showed reduced expression of T-bet transcription factors, a hallmark of Th1 cells [54]. Inversely, infected fish seem to activate Th17 cell differentiation, as suggested by simultaneous activation of the signal transducer and activator of transcription (STAT1-alpha/beta) and cytokine IL-17 [79]. Th17 cells are mainly dedicated to controlling bacterial and fungal entry [80]. In line with previous work [54, 81], our results suggest a complex orchestration of T-cell differentiation via antigen communication and associated cytokine regulatory network in *Platax* during *T. maritimum* infection. However, we cannot rule out the possibility that changes in transcript abundance might also be indicative of cell migration. Complementary approaches, including cellular imaging [82] would clarify the presence of T-reg cells in fish and improve our knowledge of their regulation.

### The genomic bases of resistance in *P. orbicularis*

Despite profound activation of the immune system response, infection was lethal for most of the individuals. At 96 hpi, less than 25% of the infected fish had survived the bacterial challenge. These surviving individuals did not display any skin lesions, suggesting that they resisted the *T. maritimum* penetration and/or limited initial bacterial adhesion. Considering the high bacterial concentration in the tanks during infection and the severity of the mortality event, it is very unlikely that resistant fish totally escaped contact with the pathogen. Indeed, *T. maritimum* were present in *resistant*_*96h*_ but not in *control*_*96h*_ individuals, hence, fish were able to maintain the integrity of their first barrier against pathogens. We hypothesise that differences in host genes activity, between resistant and control groups would reveal specific candidates genes involved in the inhibition pathogen multiplication and entry in resistant fish [16]. We show that PRRs, specifically a C-type lectin receptor, was up-regulated in *resistant*_*96h*_, together with a T-cell receptor and Low affinity immunoglobulin gamma Fc receptor II-like, while fibronectin-coding genes were down-regulated. These gene products are known for binding, agglutinating and neutralising bacteria [83], as well as triggering humoral immune response [84] or providing extracellular structure for pathogen adhesion through fibronectin-binding proteins [85, 86]. Our experimental design did not permit to tell whether there was a basal difference in expression in *resistant*_*96h*_ (genomic basis of resistance per se) or if the difference at 96 hpi was the result of a delayed adaptive immune response (timing of gene expression). Nonetheless, in catfish, lectin expression differs between families resistant or sensible to *Flavobacterium colummnare*, another gram-negative bacteria of the *Flavobacteriaceae* family [87]. Further longitudinal studies monitoring gene expression of resistant fish throughout the entire infection should prove useful in identifying resistance-specific responses to infection. Ideally, these studies should also simultaneously look at different immune-specific organ and tissue compartments and integrate genome-scale genetic variants (not limited to coding regions) and integrating a larger sampling size to infer putative genetic bases of resistance.

### Microbiome dynamics and host–pathogen communication

At 24 hpi, the microbiota was dramatically affected by the over-dominance of *T. maritimum*. Abundance of *T. maritimum* was evident from metabarcoding data and contributed to significantly reducing species richness in *infected*_*24h*_ fish. We went further and compared expression of *T. maritimum* in vivo (during infection) compared to in vitro, with the hypothesis that key drivers of pathogenicity would be called upon to enable the bacteria to thrive and break host defence barriers. There are at least two major challenges that *T. maritimum* must to overcome to successfully infect the host: Pathogens need 1) to compete for resources (at the intra and interspecific levels) to metabolise from the local environment, and 2) to resist the host immune responses and stressful conditions. During infection, *T. maritimum* enhances its glucan catabolic activity. Although this might only reflect differences due to changes in the local environmental conditions (host mucus and skin) and/or resource availability, it also reveals some major mechanisms explaining the success of *T. maritimum* at growing on fish skin. Among the genes involved in N-linked oligosaccharides utilization, we report several key components linking alternative food and mineral supplies and putative virulence-associated functions, such as several genes involved in specifically degrading and uptaking sialoglycan, as suggested by the activation of the PUL system, and ions, mainly iron [19]. Sialidase activity explains why *Capnocytophaga canimorsus* bursts when in contact with host cells as this allows the pathogen to mobilise sugar directly from host phagocytes [88]. Similarly, the tonB-coding gene, regularly reported as a gene relevant for pathogenicity, confers virulence on *Edwardsiella ctalurid* by making it possible to maintain growth in an iron-depleted medium [89]. In parallel, several stress resistance-related genes were activated during experimental infection, all of which are also involved in the antibiotic catalytic functions. These genes include *katA* and *katG*, coding for two catalase-peroxidases involved in resistance to reactive oxygen species (ROS) by detoxifying exogenous H_2_O_2_ produced by host macrophages as a defence mechanism [90]. Obviously, the identification of virulence-related genes cannot be limited to those differentiating in vitro versus in vivo infectious status and other actors might be involved in making *T. maritimum* pathogenic. For instance, siderophore-coding genes are constitutively expressed in vitro or during experimental infection. These genes are a determining factor of host–pathogen and pathogen–pathogen interactions in the so-called ‘race for iron’ [91–93], which suggests that *T. maritimum* is highly efficient at mobilising iron independently of the local environment.

Finally, we mostly explored expression level variations in the light of an exclusive interplay between the host and *T. maritimum*, which might be effective considering the over-dominance of *T. maritimum* in fish mucus. However, most of the infection systems report several pathogen co-occurrences and the presence and/or activity of other opportunistic pathogens that might also play an important role in host fate [15]. In infected fish, we found that so-called ‘opportunistic’ bacteria were relatively largely represented. Opportunistic bacteria, which include *V. harveyi*, are known for their pathogenicity to fish. *V. harveyi* is a ubiquitous bacterium and one of the most common pathogens inducing major disease outbreaks in fish farming [57]. The enrichment of *vibrio* ESVs in *resistant*_*96h*_ mucosal communities and the absence of obvious associated physiological changes (cortisol, mortality, skin integrity) in this group, suggest that *V. harveyi* alone is not sufficient to induce mortality in *Platax* under our specific experimental conditions and associated bacterial burden.

## Conclusions

Here we provide a comprehensive description of the interplay between host and *T. maritimum* under experimental infection conditions. Our results contribute to deciphering the complex orchestration of innate and immune responses of the host, but also suggest some promising avenues of research that could help to limit the impact of tenacibaculosis in fish farming. By taking an integrated ‘omic’ approach, we identified bacterial interactions as well as putative virulence-related genes in *T. maritimum* and candidate genes involved in fish resistance. Importantly, however, the detection of immune actors in fish and our comprehension of their regulation rely mainly on the quality of the annotation and the knowledge we have of their activity in other species, most of which are mammal model species. Consequently, further studies are now urgently needed to properly investigate and understand genetic and genomic bases of response to infection and possible resistance capacities in other non-model fish species.

## Supplementary Information

**Additional file 1.**

## Data Availability

Raw sequences have been deposited in the NCBI database under accession number (PRJNA656561). Codes are publicly available on Github repository https://github.com/jleluyer/metatranscriptomics_workflow
