# Supplementary material for: Dual RNAseq highlights the kinetics of skin microbiome and fish host responsiveness to bacterial infection
Source: Anim Microbiome. 2021 May 7;3:35. doi: 10.1186/s42523-021-00097-1 (PMC8106148; doi:10.1186/s42523-021-00097-1)
Supplement: Supplementary file 1 — Additional file 1. [file 42523_2021_97_MOESM1_ESM.zip › LeLuyer_etal.Microbiome.platax.SuppMat3_revision.docx]

Supplementary material

# Dual RNAseq highlights the kinetics of skin microbiome and fish host responsiveness to bacterial infection

J. Le Luyer^1,*^, Q. Schull^1, 2^, P. Auffret^1^, P. Lopez^1,3^, M. Crusot^1,4^, C. Belliard^1^, C. Basset^1^, Q. Carradec^5^, J. Poulain^5^ , S. Planes^6^, D. Saulnier^1^

^1.^ Ifremer, IRD, Institut Louis‐Malardé, Univ Polynésie française, EIO, F‐98719 Taravao, Tahiti, Polynésie Française

^2.^ MARBEC, Univ. Montpellier, Ifremer, IRD, CNRS, F‐34200, Sète, France

^3.^ Virologie et Immunologie Moléculaires, Institut National de la Recherche Agronomique, Université Paris-Saclay, Jouy-en-Josas, France

^4.^ Univ Polynésie française, Ifremer, IRD, Institut Louis‐Malardé, EIO, F‐98702 Fa’a, Tahiti, Polynésie Française

^5.^ Génomique Métabolique, Genoscope, Institut François Jacob, CEA, CNRS, Univ Evry, Université Paris-Saclay, 91057 Evry, France

^6.^ USR 3278 CRIOBE, EPHE-UPVD-CNRS, Univ. de Perpignan, France

**Corresponding author***: J. Le Luyer; e-mail:  Jeremy.le.luyer@ifremer.fr

**Keywords:** Microbiome – Gene expression – 16S rRNA – Nanopore – *Tenacibaculum maritimum* – Co-infection

**Supplementary Methods & Results**

## **Animal husbandry**

Fish were obtained from a mass tank spawning of 6 females and 8 males induced by desalinisation. Broodstock include wild individuals caught in French Polynesia that has been maintained at the Centre Ifremer du Pacifique (CIP) hatchery facility for seven years, under the supervision of the direction des ressources marines (DRM). Eggs were randomly distributed into six black circular fiberglass tanks of 210 L with 50 eggs.L^-1^ in order to achieve an average density of 30 larvae.L^-1^. Half of the tank were then reared in conditions that followed standard procedures implemented in the CIP facility (open water system, normal salinity around 36 psu), named “Standard” [1]. The other half was reared in conditions that were supposed to be optimal according to previous experiments, named “Recirculated”. Indeed, animals were bred in a recirculating system which was desalinated to 24 psu until day 34 where salinity was progressively raised to normal (36 psu). Moreover, commercial clay (Clay Bacter ®) was added daily at a rate of 1g/d/m^3^ per percent of hourly water renewal from day 1 to day 19, the beginning of living prey weaning period. In addition, an input of probiotic *Pseudoalteromonas piscicida B1* (local strain)*,* produced in CIP facilities (see supplementary methods), was realised daily in fish fed (0.5mL/day/tank) and in the water (0.5 ml/day/tank) at a concentration of 10^9^cfu/ml of bacterial suspension from day 1 to day 57. At the end of the larval phase, day 20, 700 fingerlings/tank were randomly kept. When they reached an average weight of 1g, they were sorted to get rid of the queue and head batch and kept at a density of 200/tank (1g/L). It is however relevant mentioning that around day 40, fish started to show a decrease of appetite and heavy mucus losses even if there was no mortality, mainly in the recirculating system. After an analysis of water flora, it was shown that *Vibrio harveyi* was present but no *T. maritimum*.

Platax larvae were fed 4 times a day with living preys (*Brachonius sp.* and *Artemia spp.*) before being weaned from day 16 to 23.

## **Metatranscriptomics and metabarcoding sequencing**

**Dual RNA-sequencing.** Mean number of PE raw reads reached 65.44 M ± 23.1 sd and 25.46 ± 4.31 sd, for *infected_24h_* and for *control_24h,_ control_96h_* and *resistant_96h_*, respectively. For *T. maritimum* in culture, mean raw reads reached 36.09 ± 16.26 sd. A mean of 82.47% ± 2.54 remained after filtering (Table S1). Proportion of non-host mapped reads in *infected_24h_* reached 30.71% ± 12 (Figure S1).

**Short-reads 16S rRNA metabarcoding sequencing.** We amplified the V4 region for 10–15 replicate individuals per condition. Mean number of PE raw reads reached 228,929.857 ± 39,633.05 sd out of which a mean of 77.01 ± 6.65 remained after filtering, denoising, merging and chimera’s removal (Table S2). We identified a total of 2,714 ESVs across the entire dataset.

**Nanopore full 16S rRNA metabarcoding sequencing.** We amplified the full 16S rRNA gene for 8 individuals randomly subsampled from the infected group 24 hpi to more precisely and accurately characterise the composition of the main skin microbes. The mean number of SE reads reached 60,019.62 ± 33,778.99 sd after pre-processing (with a minimum of 29,520 sequences).

*P. orbicularis* transcriptome reference

**Sampling, RNA extraction and sequencing.** One individual (50 dph) of *Platax orbicularis* was first sampled for pronephros, tegument, liver and intestine tissue following sampling of two other individuals’ samples for gonads (one male and one female), to build the host transcriptome. Total RNA was extracted from the TRIZOL mix, quantity/integrity and purity were validated by both Nanodrop readings (NanoDrop Technologies Inc.) and Bioanalyzer 2100 (Agilent Technologies). RNA was dried in RNA-stable solution (Thermo Fisher Scientific) following manufacturer’s recommendations and shipped at room temperature to McGill sequencing platform services (Montreal, Canada). TrueSeq v2 kit (Illumina, San 260 Diego, Ca, USA) was used to prepare mRNA depleted libraries that were multiplexed (13-14 samples by lane) and sequenced on HiSeq4000 100 bp PE sequencing device. Individuals that served for the transcriptome assembly were not included in this experiment.

**Transcriptome assembly**. A total of 382.34 M PE 100 bp raw reads (mean 63.72 M ± 7.44 sd) were filtered using Trimmomatic v0.36 [2], with minimum length (36 bp), trailing and leading thresholds of 26 and 26; respectively), implemented in Trinity v2.5.1 [3]. Read quality was assessed with FastQC v0.11.5 (https://www.bioinformatics.babraham.ac.uk/projects/fastqc/). Reads were assembled into transcripts using Trinity v2.5.1 [3] and default parameters. The raw transcriptome was then processed in order to reduce redundancy. First, open-reading frames (ORFs) for each transcript were predicted using ‘*LongOrfs*’ function implemented in Transdecoder v5.3.0 [3, 4]. Only the transcripts containing an ORF of at least 100 amino acids were conserved. Then, only the most expressed isoform for each gene with a minimum mapping rate of 0.5 transcripts per million (TPM) was conserved. Illumina adapters were screened in the transcriptome using a BLASTn (version 2.6.0) approach and adapter list (http://omicsoft.com/downloads/ngs/contamination_list/v1.txt). Reads were then mapped back onto the filtered transcriptome to evaluate individual mapping rate with BWA mem v0.7.15. For quality checks, the *de novo*transcriptome completeness was assessed with the BUSCO v3.0.2 [5] metazoan single-copy (n = 978) database. We added a final step to look for bacterial contamination by using BLASTn against the NCBI nt database (release 2018-08-27). Transcripts having a hit on Bacteria (e-value<10e^-4^) were discarded. The resulting transcriptome was then annotated using the Trinotate pipeline v3.1.1 (https://github.com/Trinotate/Trinotate.github.io) following standard guidelines. Detailed procedures for host transcriptome assembly are available in a Github repository (<https://github.com/paulineauffret/Transcriptome_platax>). Transcriptome statistics are given in Table S3.

## **Metatranscriptomic functional analysis**

**Differential expression and Gene Ontology enrichment.** We used a combination of differential expression and network analyses to explore host and pathogen changes in gene expression profiles during and post infection. The genes were then used for comparing functional differences based on GO enrichment analyses. Details of the results are given in Table S4**.**

References

1. Reverter M, Saulnier D, David R, Bardon-Albaret A, Belliard C, Tapissier-Bontemps N, et al. Effects of local Polynesian plants and algae on growth and expression of two immune-related genes in orbicular batfish (*Platax orbicularis*). Fish & Shellfish Immunology. 2016;58 Supplement C:82–8.

2. Bolger AM, Lohse M, Usadel B. Trimmomatic: a flexible trimmer for Illumina sequence data. Bioinformatics. 2014;:btu170.

3. Haas BJ, Papanicolaou A, Yassour M, Grabherr M, Blood PD, Bowden J, et al. De novo transcript sequence reconstruction from RNA-seq using the Trinity platform for reference generation and analysis. Nature Protocols. 2013;8:1494–1512.

4. Celaj A, Markle J, Danska J, Parkinson J. Comparison of assembly algorithms for improving rate of metatranscriptomic functional annotation. Microbiome. 2014;2:39.

5. Simão FA, Waterhouse RM, Ioannidis P, Kriventseva EV, Zdobnov EM. BUSCO: assessing genome assembly and annotation completeness with single-copy orthologs. Bioinformatics. 2015;31:3210–2.

6. Pérez-Pascual D, Lunazzi A, Magdelenat G, Rouy Z, Roulet A, Lopez-Roques C, et al. The Complete Genome Sequence of the Fish Pathogen *Tenacibaculum maritimum* Provides Insights into Virulence Mechanisms. Frontiers in Microbiology. 2017;8.

**Supplementary Figures & Tables**

Figure S1: Proportion of reads mapped to the microbial compartment. Read origin was dissociated *in silico*. Reads were considered to originate from the microbial compartment when no mapping was apparent in the fish transcriptome. Ctl-24h: *control_24h_*; Ctl-96h: *control_96h_*, Inf-24h: *infected_24h_*; Res-96h: *resistant_96h_*, groups. Different letters indicate significant differences, *P* < 0.05, Dunn’s test.

Figure S2: Genetic variation and relatedness across established phenotypes. Principal component analysis for the total filtered dataset including 13,448 bi-allelic markers.

**
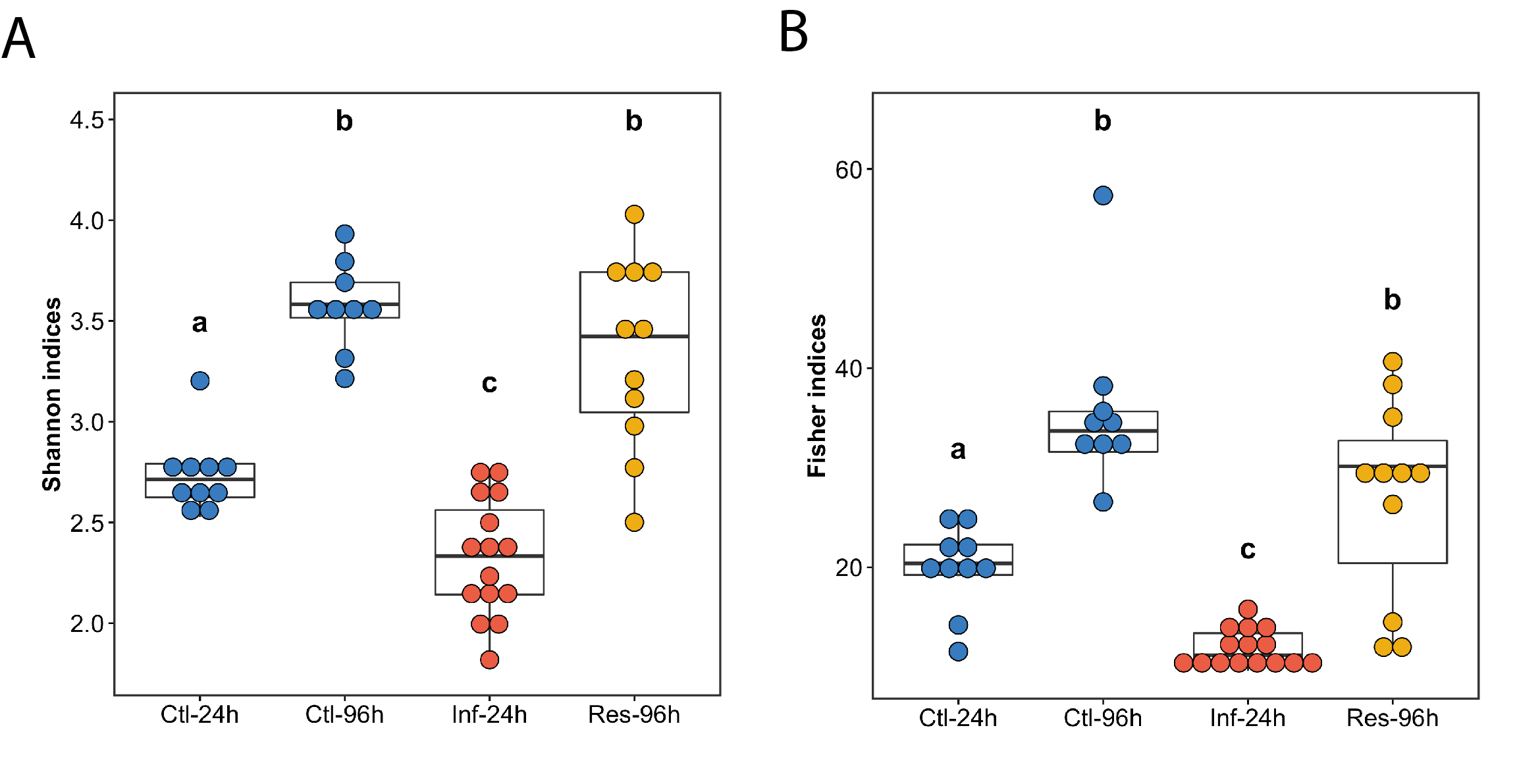
**

Figure S3: Alpha-diversity estimates across groups. Alpha diversity was computed using A) Shannon (H’) and B) Fisher indexes. Ctl-24h: *control_24h_*; Ctl-96h: *control_96h_*, Inf-24h: *infected_24h_*; Res-96h: *Resistant_96h_*, groups. Different letters indicate significant differences, *P* < 0.05, Tukey’s HSD. Each dot represents a single individual.


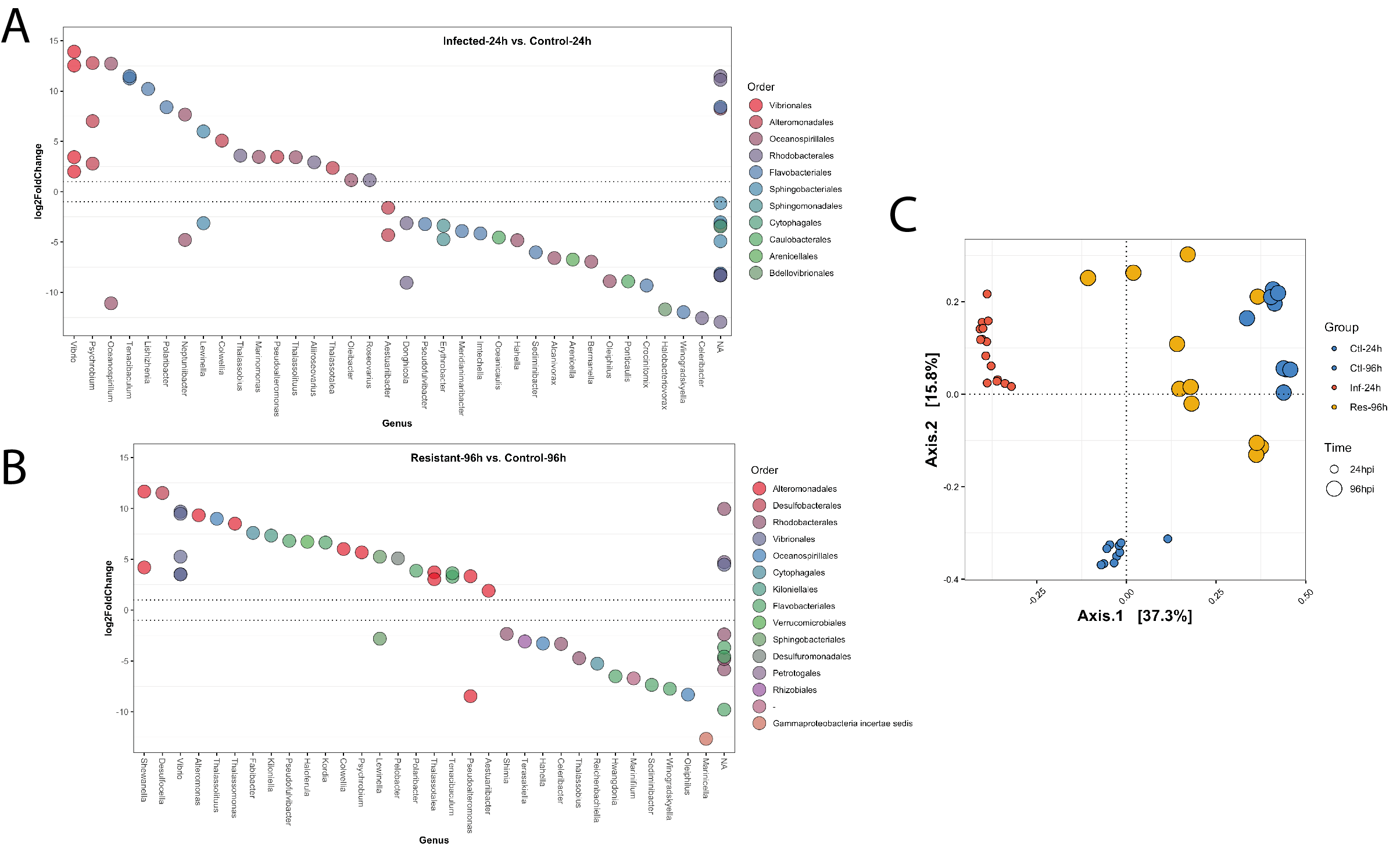


Figure S4: Taxa enrichment and beta-diversity dissimilarities across groups. A) ASV enrichment between *infected_24h_* (positive log2FC) and *control_24h_* (negative values). Colours represent different orders. The y-axis reports shrunken log2FC. Horizontal dash lines represent the log2FC threshold for significance (|log2FC| > 2). Nas represent missing taxonomic information for this ASV; B) ASV enrichment between *resistant_96h_* (positive log2FC) and *control_96h_* (negative values). Colours represent different orders. The x-axis represents associated genera; the y-axis reports shrunken log2FC. Nas represent missing taxonomic information for this ASV. C) PcoA based on Bray-Curtis distance values. Data were transformed in proportions as recommended for Bray-Curtis distances. Ctl-24h: *control_24h_*; Ctl-96h: *control_96h_*, Inf-24h: *infected_24h_*; Res-96h: *Resistant_96h_*, groups. Smaller points code for 24 hpi, larger points for 96 hpi groups.

Figure S5: Relative abundance of bacterial genera in the *infected_24h_* group reported with Nanopore. 16S rRNA sequences and their relative abundance were generated from Nanopore reads (see methods). These sequences were blasted against the NCBI nt database (BLASTn; e-value<10-5). Dot size represents the number of unique sequences per genus.

Figure S6: Total Illumina PE read counts for the most abundant species in the microbial compartment. The selected reference species were the most abundant species represented in the Nanopore 16S rRNA analysis (see methods). T.mar: *T. maritimum*; V.har: *Vibrio harveyi*; A.medit: *Altermonas mediteranea*; P.phe: *Pseudoalteromonas phenolytica*; V.algi: *Vibrio alginolyticus*; and S.yano: *Sphingobium yanoikuyae*. Each dot per species represents one individual.

Figure S7: Plot of expression levels of in vitro and in vivo groups for all virulence-related genes previously identified. The gene list was obtained from whole-genome analysis in *Tenacibaculum* spp. [6]. Asterisks indicate genes with a significant difference between groups (Shrunken |log2FC| > 2; FDR < 0.01).

Figure S8: Genes surrounding the susC-susD pair in *T. maritimum* genome. Numbers on the top represent the different genes with : 1) MARIT_RS12870: SusC/RagA/ family, TonB-dependent receptor; 2) MARIT_RS12875: SusD/RabgB family Lipoprotein precursor; 3) MARIT_RS12880: N-acetylneuraminate lyase; 4) MARIT_RS12885: Potential Transcriptional Regulator fadR family; 5) MARIT_RS12890: N-acetyl-D-glucosamine 2-epimerase; 6) MARIT_RS12895: Potential hydrolase/lipase; 7) MARIT_RS12900: Creatinase family protein; 8) MARIT_RS12905: SiaA/NanH, Multimodular sialidase/Sialate O-acetylesterase/sialidase, family GH33 containing a C-terminal secretion signal; and 9) MARIT_RS12910: NanN/NanT, Symporter of sialic acid. Red color indicates genes significantly up-regulated in infection condition for *T. maritimum* (Shrunken |log2FC| > 4; FDR < 0.01). A fold change ratio of around 2 was found for MARIT_RS12885 in infection condition for *T. maritimum (Fig. S7).*

Table S1: Individual mapping statistics against a combination of the *P. orbicularis* transcriptome and bacterial genomes. Transcriptome column refers to samples included in the metatranscriptome assembly. hpi = hours post-infection. PE = paired end. Mapping rate values are computed based on mapping against the combined reference transcriptome (host + microbiome).

Table S2: Individual sequencing and mapping statistics for MiSeq Illumina reads. hpi = hours post-infection. Denoised_Fwd and Denoised_Fwd represent the number of reads after denoised for the forward and reverse reads, respectively.

Table S3: *P. orbicularis* transcriptome statistics.

Table S4: List of Fish and *T. maritimum* DEGs and GO term enrichments

Excel file: LeLuyer_etal_microbiome.TabS4.xls
